# Relationship between metabolically unhealthy obesity and chronic obstructive pulmonary disease among U.S. adults: Evidence from NHANES 1999–2018

**DOI:** 10.1016/j.clinsp.2026.101006

**Published:** 2026-07-17

**Authors:** Shuang Li, Xing Fang, Pingfei Wang, Huasheng Wei

**Affiliations:** a School of Medicine and Life Sciences, Chengdu University of TCM, Chengdu, Sichuan, China; b Department of Respiratory and Critical Care Medicine, Dazhou Central Hospital, Dazhou, Sichuan, China

**Keywords:** National health and nutrition examination survey, Chronic obstructive pulmonary disease, Metabolically healthy obesity

## Abstract

**Background:**

This research aimed to examine the association of Metabolically Unhealthy Obesity (MUO) with the prevalence of Chronic Obstructive Pulmonary Disease (COPD).

**Methods:**

The present research applied a cross-sectional design, and participants from the National Health and Nutrition Examination Survey 1999–2018 were enrolled. The association between metabolically healthy and unhealthy obesity phenotypes and COPD was examined through multivariable logistic regression analyses.

**Results:**

In total, 28,401 individuals were enrolled, among whom 2403 had COPD. After controlling for confounders, Body Mass Index (BMI) exhibited a positive association with the prevalence of COPD (OR = 1.04, 95% CI: 1.03–1.05, p < 0.001). MUO was associated with significantly elevated prevalence of COPD compared with Metabolically Healthy Normal Weight (MH—NW) (OR = 2.02, 95% CI 1.51‒2.70). BMI was linearly associated with COPD in the metabolically healthy group (P-non-linear = 0.276), whereas in the metabolically unhealthy group, the association was nonlinear (P-non-linear = 0.002). According to receiver operating characteristic curve analysis, the Area Under the Curve (AUC) of BMI for diagnosing COPD was 0.735 (95% CI 0.695–0.775) in metabolically healthy individuals and 0.747 (95% CI 0.736–0.757) in metabolically unhealthy individuals.

**Conclusion:**

MUO is associated with a higher prevalence of COPD in U.S. adults. Regardless of metabolic health status, obesity is associated with a higher prevalence of COPD. These findings suggest that maintaining a healthy body weight through lifestyle interventions may be beneficial across all populations, even providing additional benefits among metabolically healthy individuals.

## Highlights

• MUO in U.S. adults exhibits increased COPD risk relative to MH—NW.
• Obesity, irrespective of metabolic health, augments COPD rates: 1999‒2018 NHANES data.
• BMI linearly correlates with COPD risk in metabolically healthy individuals yet nonlinearly in unhealthy.
• Necessary lifestyle changes to manage weight for COPD prevention in all demographics.

## Introduction

Chronic Obstructive Pulmonary Disease (COPD) stands as one of the top three major causes of mortality across the globe, with its incidence particularly high in middle- and low-income countries, accounting for up to 90% of cases.^1^ COPD is a critical public health issue, yet it is largely preventable. Globally, COPD represents a major cause of chronic disease morbidity and death. A large number of patients experience long-term health impairments and premature death due to COPD and its complications. Given the aging population and the ongoing exposure to factors influencing the risk of COPD, the disease burden of COPD is projected to rise in the next few decades.^2^ Hence, identifying relevant risk markers and designing effective preventive approaches are essential to decrease the individual and socioeconomic burden associated with COPD.

During the past fifty years, obesity prevalence has risen dramatically. Existing evidence demonstrates that it correlates with a heightened risk of numerous diseases, encompassing stroke, Cardiovascular Disease (CVD), as well as multiple malignancies.^3^ In addition, obesity correlates with a spectrum of respiratory diseases, encompassing asthma, obstructive sleep apnea, pulmonary embolism,^4^ and COPD.^5^ Beyond the established risk makers for COPD, obesity may also act as a potential contributor to COPD development.^6^

Obesity is closely correlated with metabolic abnormalities, and metabolic disturbances related to obesity are considered mediators in the development of obesity-related comorbidities.^7^ Notably, some individuals with obesity have few or no metabolic abnormalities, a phenotype referred to as Metabolically Healthy Obesity (MHO).^8^^,^^9^ Metabolic heterogeneity exists among obese populations, and metabolic dysfunction is correlated with the onset and progression of COPD.^10^^,^^11^ Accordingly, further stratification of overweight and obese populations according to metabolic health status is necessary to disentangle the complex interplay between overweight/obesity and metabolic disturbances.

To date, systematic research examining the association between metabolically defined obesity or overweight phenotypes and COPD remains scarce. According to data derived from the National Health and Nutrition Examination Survey (NHANES), the authors aimed to estimate the association of different metabolic health phenotypes with COPD prevalence. Through this analysis, the authors aimed to identify modifiable factors that may inform clinical prevention and the development of intervention approaches.

## Methods

### Data collection

The original data were obtained from the NHANES. Through a complex, multistage, and stratified sampling design, this survey collects nutritional and health data every two years from a nationally representative sample of the non-institutionalized population (https://www.cdc.gov/nchs/nhanes/). The NHANES data are de-identified and publicly available; therefore, this secondary analysis was exempt from institutional ethics review.

**Fig. 1:**
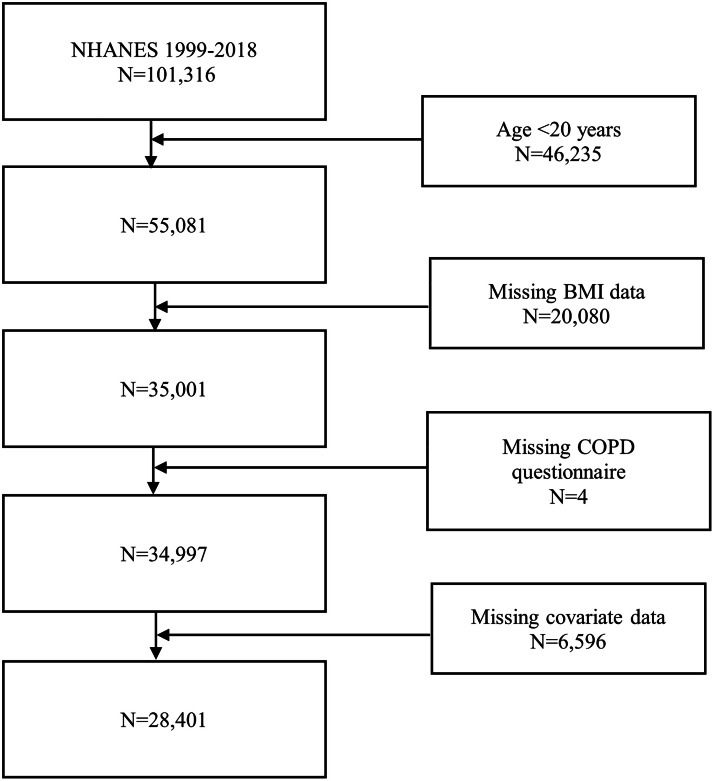
Flowchart of NHANES 1999–2018 sample selection.

Data from ten continuous NHANES cycles spanning 1999 to 2018 were utilized in this study. Exclusion criteria included: (i) Individuals younger than 20-years; (ii) Individuals without available Body Mass Index (BMI) measurements or assessments of metabolic health status; (iii) Individuals who not able to complete questionnaires related to COPD; and (iv) Individuals with missing information on major variables, encompassing marital status, sex, educational attainment, age, dietary factors, and race. After applying these criteria, 28,401 participants were enrolled (Fig. 1).

### Definition of MHO and COPD

Weight status was categorized based on BMI, measured as weight in kilograms divided by height in meters squared. Based on U.S. guidelines,^12^ BMI categories were defined as obesity (at or above 30 kg/m^2^), overweight (between 25.0 and 29.9 kg/m^2^), as well as normal weight (between 18.5 and 24.9 kg/m^2^). Individuals were confirmed as metabolically unhealthy if they exhibited at least one metabolic abnormality, encompassing (i) Hypertension: self-reported diagnosis; use of antihypertensive medication; or measured diastolic blood pressure at or above 85 mmHg or systolic blood pressure at or above 130 mmHg; (ii) Diabetes: self-reported diagnosis; use of hypoglycemic medication or insulin; hemoglobin A1C at or above 5.7%; two-hour oral glucose tolerance test at or above 140 mg/dL; or fasting glucose at or above 100 mg/dL; (iii) Abnormal High-Density Lipoprotein Cholesterol (HDL-C) levels: HDL-C below 50 mg/dL for females and HDL-C below 40 mg/dL for males; (iv) Current administration of lipid-lowering medications or low-density lipoprotein >150 mg/dL. Accordingly, six metabolic health phenotypes were defined based on weight status: Metabolically Healthy Overweight (MH—OW), MHO, Metabolically Unhealthy Overweight (MU-OW), Metabolically Healthy Normal Weight (MH—NW), Metabolically Unhealthy Normal Weight (MU-NW), as well as Metabolically Unhealthy Obesity (MUO).^13^

Individuals were considered to have COPD if they met one or more of these criteria.^14^ (i) A post-bronchodilator ratio of forced expiratory volume in one second to forced vital capacity under 0.70 after inhaling a β2-adrenergic bronchodilator; (ii) The participant had been informed by a physician or other health professional that they had emphysema; (iii) Participants aged 40-years or above with documented histories of chronic bronchitis and smoking, and had received medications including inhaled corticosteroids, mast cell stabilizers, selective phosphodiesterase-4 inhibitors, or leukotriene modifiers. Those who did not meet the diagnostic criteria were confirmed as non-COPD.

### Covariates

Several covariates were included, encompassing educational attainment (above high school, below high school, or high school or equivalent), sex (woman or man), alcohol consumption, race (non-Hispanic Black, Mexican American, other Hispanic, non-Hispanic White, and other races), Poverty-to-Income Ratio (PIR), age (45-years or older, between 45- and 60-years, older than 60-years), CVD status, and smoking status. Dietary nutrient intake, encompassing total fat, carbohydrates, protein, and dietary fiber, was also assessed. These variables were self-reported via standardized questionnaires administered during household interviews. Alcohol consumption was categorized according to whether participants reported consuming >12 alcoholic drinks within the past year. Smoking status was classified based on participants’ binary response to whether they had smoked at least 100 cigarettes over their lifetime. Those who gave a negative response were categorized as never smokers, whereas those who gave an affirmative response were further classified as current or former smokers, depending on whether they were still smoking. Based on relevant information from participants’ medical history, the presence of CVD was determined as absent or present. Dietary nutrient intake was analyzed as a continuous variable to examine the influence of different nutrient intake levels on health outcomes. The full definitions and data collection methods for covariates are available in the technical documentation and user guides provided on the NHANES website.

### Statistical analysis

An in-depth analysis of weighted samples was carried out to correct for potential deviations in survey representativeness of the overall U.S. adults, thereby enhancing the generalizability of the data. Mean ± standard deviation was used to represent normally distributed continuous variables, while median with interquartile range was used to represent non-normally distributed continuous variables. Inter-group comparisons among normally distributed variables were performed via the Student’s *t*-test, while group differences among non-normally distributed variables were assessed by nonparametric tests. Categorical variables were represented by weighted counts (weighted percentages), and the Chi-Square test was applied to make inter-group comparisons. Multicollinearity among covariates was examined using the Variance Inflation Factor (VIF). All VIF values were below five, suggesting no significant multicollinearity. Five logistic regression models were built to assess the relationships between BMI and Metabolically Healthy and Unhealthy Obesity (MHO and MUO) phenotypes and COPD. Model 1 was unadjusted. Model 2 was adjusted for demographic factors (age, sex, race, and education). Model 3 was adjusted for demographics and lifestyle factors (marital status, PIR, alcohol, and smoking). Model 4 was additionally adjusted for CVD, on the basis of Model 3. Model 5 was additionally adjusted for dietary intake, on the basis of Model 4. Model 3 served as the main explanatory model. The relationships between BMI, MHO and MUO phenotypes, and the odds of COPD were examined using Odds Ratios (ORs) and 95% Confidence Intervals (95% CIs). A potential nonlinear association between BMI and COPD among metabolically healthy and unhealthy individuals was estimated through Restricted Cubic Spline (RCS) analyses. These analyses could provide insight into the complex association between BMI and COPD. Receiver Operating Characteristic (ROC) curves, an effective tool for assessing the diagnostic performance of predictive models, were utilized to appraise the accuracy of BMI in assessing the odds of COPD among metabolically healthy and unhealthy individuals. To test the stability of the findings, Multiple sensitivity analyses based on Propensity Score Matching (PSM) were carried out to balance confounding bias from covariates, and the main results were subsequently validated. Secondly, the authors excluded individuals with a history of CVD, and further redefined metabolically healthy obesity using only biochemical indicators. After excluding self-reported diagnoses and medication use, the authors repeated this analysis. Finally, individuals with hypertension and diabetes were excluded to verify the stability of the results. R software (version 4.5.0) was applied to carry out statistical analyses, and a two-sided p-value below 0.05 was regarded as statistically significant.

## Results

There were 28,401 eligible individuals included in this study, among whom 2403 had COPD. Among them, 1094 people had abnormal lung function; 646 people were informed of the diagnosis of emphysema; and 169 people received medication treatment. (Table 1). The baseline characteristics of all included individuals are presented in Table 1. The overall mean BMI was 29.14 kg/m^2^. The sex distribution was relatively balanced, with 51.24% female and 48.76% male. Age distribution was as follows: ≤45-years, 43.23%; 45–60 years, 30.16%; > 60-years, 26.61%. Regarding educational attainment, 14.86% of included individuals had attained less than a high school education, 61.53% had an education above high school, and 23.61% had a high school diploma or equivalent. The study population was racially and ethnically diverse: 5.00% other Hispanic, 9.68% non-Hispanic Black, 7.06% Mexican American, 72.04% non-Hispanic White, and 6.22% other races. Regarding marital status, 19.09% of individuals included were divorced, widowed, or separated, 14.81% had never married, and 66.10% were married or living with a partner. Regarding alcohol consumption, 73.50% reported drinking, while 26.50% reported no alcohol intake. Analysis of smoking status indicated that 27.43% were former smokers, 52.89% were non-smokers, and 19.68% were current smokers. Metabolic health status based on MHO, MUO, and related phenotypes demonstrated significant heterogeneity in distribution, with MUO being the most prevalent (33.84%). Compared with individuals without COPD, the proportion of women was markedly elevated (60.87%vs. 50.37%), and the proportion of males was notably lower (39.13%vs. 49.63%) among individuals with COPD. In comparison with individuals without COPD, those with COPD exhibited a markedly elevated mean BMI (30.77 vs. 28.99 kg/m^2^). Additionally, CVD prevalence was markedly higher among individuals with COPD than those without COPD (26.40%vs. 8.48%). In terms of dietary intake, individuals with COPD consumed significantly lower amounts of protein (74.90 vs. 83.77 g), carbohydrates (243.84 vs. 258.61 g), fat (79.33 vs. 85.07 g), and fiber (14.53 vs. 16.95 g) in comparison with individuals without COPD. All differences were statistically significant (p < 0.05, Table 1).

**Table 1:** Baseline characteristics of participants according to COPD.

Characteristic	Overall	Normal	COPD	p-value
BMI (kg/m^2^)	29.14 (6.76)	28.99 (6.62)	30.77 (7.92)	<0.001
Gender				<0.001
Female	14,470 (51.24%)	13,095 (50.37%)	1375 (60.87%)	
Male	13,931 (48.76%)	12,903 (49.63%)	1028 (39.13%)	
Age				<0.001
≤ 45	11,181 (43.23%)	10,641 (44.79%)	540 (25.98%)	
45‒60	7252 (30.16%)	6600 (30.08%)	652 (31.09%)	
> 60	9968 (26.61%)	8757 (25.14%)	1211 (42.93%)	
PIR	3.11 (1.62)	3.16 (1.61)	2.57 (1.59)	<0.001
Education				<0.001
< High school diploma	6733 (14.86%)	6062 (14.30%)	671 (21.14%)	
> High school diploma	15,139 (61.53%)	14,029 (62.50%)	1110 (50.76%)	
High school diploma/equivalent	6529 (23.61%)	5907 (23.21%)	622 (28.10%)	
Race				<0.001
Mexican American	4512 (7.06%)	4334 (7.46%)	178 (2.65%)	
Non-Hispanic Black	5581 (9.68%)	5168 (9.82%)	413 (8.13%)	
Non-Hispanic White	13,567 (72.04%)	12,055 (71.32%)	1512 (79.96%)	
Other Hispanic	2265 (5.00%)	2113 (5.13%)	152 (3.51%)	
Other Race	2476 (6.22%)	2328 (6.26%)	148 (5.75%)	
HDL (mmoL/L)	53.43 (16.28)	53.52 (16.14)	52.34 (17.63)	0.003
Marital				<0.001
Married/Living with partner	17,563 (66.10%)	16,287 (66.75%)	1276 (58.90%)	
Never married	4261 (14.81%)	3993 (15.20%)	268 (10.42%)	
Widowed/ Divorced/ Separated	6577 (19.09%)	5718 (18.05%)	859 (30.68%)	
Drinking				<0.001
No	8940 (26.50%)	8142 (26.15%)	798 (30.31%)	
Yes	19,461 (73.50%)	17,856 (73.85%)	1605 (69.69%)	
Smoke				<0.001
Current smoker	5549 (19.68%)	4748 (18.28%)	801 (35.18%)	
Former smoker	7829 (27.43%)	6939 (26.74%)	890 (35.06%)	
Never smoker	15,023 (52.89%)	14,311 (54.98%)	712 (29.76%)	
CVD				<0.001
No	24,791 (90.04%)	23,121 (91.52%)	1670 (73.60%)	
Yes	3610 (9.96%)	2877 (8.48%)	733 (26.40%)	
Protein	83.04 (41.68)	83.77 (41.80)	74.90 (39.43)	<0.001
CHO	257.39 (124.11)	258.61 (124.23)	243.84 (122.01)	<0.001
Fat	84.60 (46.21)	85.07 (46.32)	79.33 (44.64)	<0.001
Fiber	16.75 (10.31)	16.95 (10.39)	14.53 (9.12)	<0.001
MHO				<0.001
MH—NW	1853 (8.17%)	1774 (8.53%)	79 (4.14%)	
MH—OW	1304 (5.54%)	1258 (5.80%)	46 (2.67%)	
MHO	940 (3.73%)	881 (3.79%)	59 (3.11%)	
MU-NW	5899 (20.31%)	5411 (20.41%)	488 (19.14%)	
MU-OW	8427 (28.41%)	7790 (28.62%)	637 (26.12%)	
MUO	9978 (33.84%)	8884 (32.85%)	1094 (44.82%)	

BMI, Body Mass Index; PIR, Poverty and Income Ratio; CVD, Cardiovascular Disease; CHO, Carbohydrate; HDL, High-Density Lipoprotein; MH—NW, Metabolism Healthy Normal Weight; MH—OW, Metabolism Healthy Overweight; MHO, Metabolism Healthy Obesity; MU-NW, Metabolism Unhealthy Normal Weight; MU-OW, Metabolism Unhealthy Overweight; MUO, Metabolism Unhealthy Obesity.

Table 2 presents the association between BMI and COPD examined by the multivariable logistic regression analyses. BMI was analyzed both as a categorical and continuous variable across five progressively adjusted models. All five models suggested a significantly positive association between BMI and the prevalence of COPD (Model 1: OR = 1.04, 95% CI 1.03–1.04; Model 2: OR = 1.04, 95% CI 1.03–1.04; Model 3: OR = 1.04, 95% CI 1.03–1.05; Model 4: OR = 1.04, 95% CI 1.03–1.04; Model 5: OR = 1.04, 95% CI 1.03–1.04). BMI was further stratified into subgroups, with the normal-weight group serving as the reference. The results demonstrated that obesity was associated with significantly elevated prevalence of COPD among five models (Model 1: OR = 1.63, 95% CI 1.42–1.84; Model 2: OR = 1.62, 95% CI 1.41–1.85; Model 3: OR = 1.72, 95% CI 1.50–1.96; Model 4: OR = 1.64, 95% CI 1.43–1.87; Model 5: OR = 1.62, 95% CI 1.42–1.85).

**Table 2:** Relationship between BMI and COPD.

Participants	Model 1	Model 2	Model 3	Model 4	Model 5
	OR (95% CI)	OR (95% CI)	OR (95% CI)	OR (95% CI)	OR (95% CI)
BMI					
Continuous	1.04 (1.03‒1.04)^a^	1.04 (1.03‒1.04)^a^	1.04 (1.03‒1.05)^a^	1.04 (1.03‒1.04)^a^	1.04 (1.03‒1.04)^a^
Grouping					
Normal weight	Ref.	Ref.	Ref.	Ref.	Ref.
Overweight	1.04 (0.89‒1.22)	1.04 (0.89‒1.22)	1.11 (0.96‒1.29)	1.11 (0.95‒1.28)	1.10 (0.95‒1.28)
Obesity	1.63 (1.42‒1.84)^a^	1.62 (1.41‒1.85)^a^	1.72 (1.50‒1.96)^a^	1.64 (1.43‒1.87)^a^	1.62 (1.42‒1.85)^a^

Note: ‘^a^’ 0.001 ‘^⁎⁎^’ 0.01 ‘*’ 0.05.

Model 1, NO covariate has been adjusted. Model 2: Adjusted Gender + Age grouping + Race + Education. Model 3: Adjusted Gender + Age grouping + Race + Education + Marital + PIR + Drinking + Smoke. Model 4: Adjusted Gender + Age grouping + Race + Education + Marital + PIR + Drinking + Smoke + CVD. Model 5: Adjusted Gender + Age grouping + Race + Education + Marital + PIR + Drinking + Smoke + CVD + protein + CHO + Fat + Fiber.

As illustrated in Table 3, the association between MHO and MUO phenotypes and COPD was further assessed. In the primary adjusted model (Model 3), MUO was associated with a significantly higher prevalence of COPD (OR = 2.02, 95% CI 1.51‒2.70) compared to MH—NW. This association remained robust in sensitivity analyses after adjusting for CVD (Model 4) and dietary factors (Model 5).

**Table 3:** Relationship between metabolically healthy obesity and COPD.

Participants	Model 1	Model 2	Model 3	Model 4	Model 5
	OR (95% CI)	OR (95% CI)	OR (95% CI)	OR (95% CI)	OR (95% CI)
MHO					
MH—NW	Ref.	Ref.	Ref.	Ref.	Ref.
MH—OW	0.95 (0.57‒1.57)	0.95 (0.58‒1.55)	1.03 (0.63‒1.68)	1.06 (0.65‒1.73)	1.07 (0.66‒1.75)
MHO	1.69 (1.07‒2.68)	1.75 (1.11‒2.75)^c^	1.75 (1.11‒2.74)^c^	1.73 (1.10‒2.70)^c^	1.70 (1.09‒2.64)^c^
MU-NW	1.93 (1.45‒2.58)^a^	1.41 (1.05‒1.89)^c^	1.21 (0.92‒1.60)	1.17 (0.89‒1.55)	1.17 (0.89‒1.54)
MU-OW	1.88 (1.41‒2.51)^a^	1.44 (1.06‒1.94)^c^	1.34 (1.00‒1.79)^c^	1.28 (0.96‒1.72)	1.28 (0.96‒1.71)
MUO	2.82 (2.10‒3.77)^a^	2.16 (1.61‒2.91)^a^	2.02 (1.51‒ 2.70)^a^	1.87 (1.41‒ 2.50)^a^	1.86 (1.39‒ 2.47)^a^

Note: ‘^a^’ 0.001 ‘**’ 0.01 ‘^c^’ 0.05.

Model 1: NO covariate has been adjusted. Model 2: Adjusted Gender + Age grouping + Race + Education. Model 3: Adjusted Gender + Age grouping + Race + Education + Marital + PIR + Dringking + Smoke. Model 4: Adjusted Gender + Age grouping + Race + Education + Marital + PIR + Drinking + Smoke + CVD. Model 5: Adjusted Gender + Age grouping + Race + Education + Marital + PIR + Drinking + Smoke + CVD + protein + CHO + Fat + Fiber. MH—NW, Metabolism Healthy Normal Weight; MH—OW, Metabolism Healthy Overweight; MHO, Metabolism Healthy Obesity; MU-NW, Metabolism Unhealthy Normal Weight; MU-OW, Metabolism Unhealthy Overweight; MUO, Metabolism Unhealthy Obesity.

Participants were stratified into metabolically healthy and metabolically unhealthy subgroups. A potential nonlinear association between BMI and COPD was examined through RCS analysis. The diagnostic accuracy of BMI for COPD was appraised by ROC curves.

**Fig. 2:**
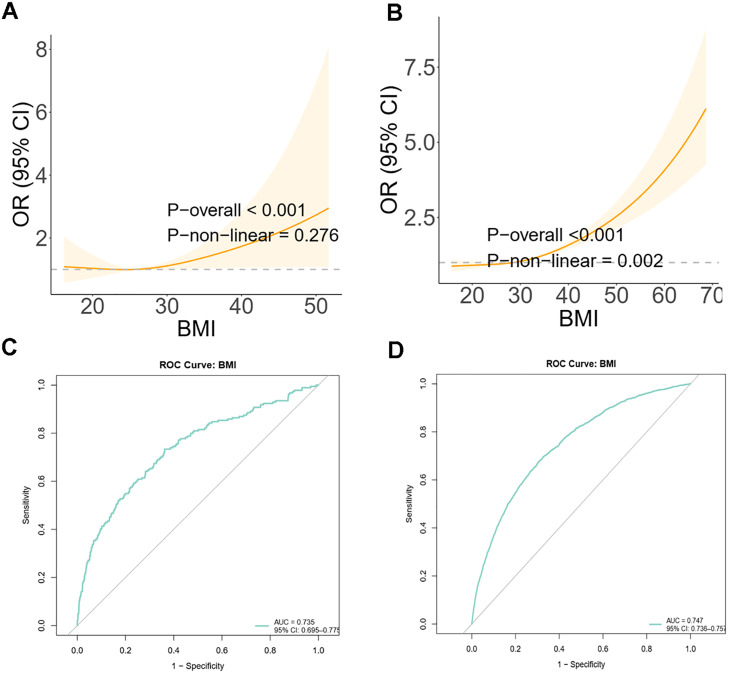
Participants were stratified into metabolically healthy and metabolically unhealthy subgroups. (A) The association between BMI and COPD in healthy metabolomic groups; (B) The association between BMI and COPD in unhealthy metabolomic groups; (C) In metabolically healthy groups, the area under the AUC for BMI; (D) In metabolically unhealthy groups, the area under the AUC for BMI.

As shown in Fig. 2A, a linear association was found between BMI and the prevalence of COPD in metabolically healthy individuals (P for non-linearity = 0.276). The prevalence of COPD increased consistently across BMI levels, without evidence of threshold effects. As illustrated in Fig. 2B, a non-linear association was found between BMI and the prevalence of COPD in metabolically unhealthy individuals (P for non-linearity = 0.002), with a turning point around the upper-overweight range. Fig. 2C shows the diagnostic performance of BMI for COPD in metabolically healthy individuals. The Area Under the Curve (AUC) was 0.735 (95% CI 0.695–0.775), indicating moderate discriminatory performance of BMI for COPD in metabolically healthy individuals. Fig. 2D presents the ROC analysis for metabolically unhealthy individuals. The AUC was higher, at 0.747 (95% CI 0.736–0.757), suggesting slightly better diagnostic accuracy in this subgroup.

Table 4 presents the detailed associations between COPD and MHO and MUO phenotypes. Subgroup analyses stratified by education level, sex, smoking status, race, marital status, age, and alcohol consumption were conducted. The results demonstrated that, using the MH—NW group as reference, the MUO was significantly associated with COPD in both females (OR = 1.93, 95% CI 1.35–2.78) and males (OR = 1.79, 95% CI 1.12–2.86, p < 0.05). Significant associations were also found in individuals aged < 45-years and 45–60 years (OR = 1.99, 95% CI 1.37–2.88; OR = 2.18, 95% CI 1.11–4.30, respectively; p < 0.05). Further analyses demonstrated that the association between MUO and COPD was more significant in those aged ≤ 45-years, non-Hispanic Whites, those receiving higher education, married or cohabiting individuals, alcohol consumers, and former smokers, with corresponding ORs of 1.99 (95% CI 1.37–2.88), 1.78 (95% CI 1.27–2.50), 2.31 (95% CI 1.57–3.41), 2.44 (95% CI 1.56–3.81), 1.95 (95% CI 1.36–2.79), and 2.92 (95% CI 1.75–4.86), all statistically significant (p < 0.001, Table 4). These factors may be positively related to the prevalence of COPD. Significant interactions were found for marital status (P for interaction = 0.031), education level (P for interaction = 0.026), and age (P for interaction = 0.031).

**Table 4:** The association between COPD and metabolically healthy obesity according to different variables.

Subgroup	OR (95% CI)						P for interaction
	MH—NW	MH—OW	MHO	MU-NW	MU-OW	MUO	
**Gender**							0.399
Female	**Ref**	1.27 (0.71‒2.27)	1.89 (1.11‒3.22)*	1.12 (0.79‒1.58)	1.29 (0.89‒1.86)	1.93 (1.35‒2.78)a	
Male	**Ref**	0.84 (0.36‒1.94)	1.36 (0.62‒2.99)	1.29 (0.80‒2.07)	1.30 (0.82‒2.08)	1.79 (1.12‒2.86)^c^	
**Age**							0.031
≤ 45	**Ref**	0.88 (0.47‒1.63)	1.23 (0.68‒2.22)	0.79 (0.52‒1.19)	1.37 (0.90‒2.09)	1.99 (1.37‒2.88)^a^	
45‒60	**Ref**	0.89 (0.29‒2.77)	2.64 (1.06‒6.54)	1.52 (0.79‒ 2.94)	1.36 (0.69‒2.69)	2.18 (1.11‒4.30)^c^	
> 60	**Ref**	2.00 (0.58‒6.85)	1.84 (0.56‒ 6.08)	1.24 (0.63‒2.44)	1.20 (0.59‒2.43)	1.57 (0.78‒3.15)	
**Race**							0.742
Mexican American	**Ref**	0.95 (0.21‒4.33)	1.11 (0.29‒4.18)	0.76 (0.25‒2.28)	1.00 (0.36‒2.81)	1.36 (0.48‒3.84)	
Non-Hispanic Black	**Ref**	1.15 (0.50‒2.64)	1.24 (0.50‒3.04)	1.32 (0.62‒2.80)	1.31 (0.63‒2.72)	1.88 (0.93‒3.82)	
Non-Hispanic White	**Ref**	1.09 (0.61‒1.94)	1.81 (1.05‒3.11)	1.13 (0.82‒1.56)^c^	1.26 (0.89‒1.76)	1.78 (1.27‒2.50)^a^	
Other Hispanic	**Ref**		1.30 (0.23‒7.35)	0.91 (0.20‒4.16)	1.04 (0.23‒4.61)	1.40 (0.33‒6.02)	
Other Race	**Ref**	2.72 (0.52‒14.1)	1.35 (0.21‒8.58)	2.21 (0.68‒7.21)	1.95 (0.57‒6.71)^c^	4.37 (1.36‒14.1)	
**Education**							0.026
< High school diploma	**Ref**	0.73 (0.27‒1.95)	1.01 (0.39‒2.64)	0.81(0.45‒1.48)	0.64 (0.34‒1.21)	1.00 (0.52‒1.90)	
> High school diploma	**Ref**	0.97 (0.51‒1.86)	2.20 (1.26‒3.85)^b^	1.13 (0.75‒1.72)	1.61 (1.09‒2.38)^c^	2.31 (1.57‒3.41)^a^	
High school diploma/ Equivalent	**Ref**	1.72 (0.70‒4.26)	1.34 (0.53‒3.35)	1.45 (0.76‒2.75)	1.37 (0.71‒2.67)	1.86 (0.98‒3.52)	
**Marital**							0.031
Married/Living with partner	**Ref**	1.77 (0.92‒3.42)	2.63 (1.40‒4.92)^b^	1.56 (1.00‒2.45)	1.704 (1.10‒2.78)^c^	2.44 (1.56‒3.81)^a^	
Never married	**Ref**	0.47 (0.13‒1.64)	0.67 (0.27‒1.69)	0.56 (0.30‒1.05)	1.08 (0.55‒2.13)	1.60 (0.88‒2.94)	
Widowed/ Divorced/ Separated	**Ref**	0.42 (0.16‒1.12)	1.38 (0.56‒3.37)	1.02(0.60‒1.71)	0.93(0.55‒1.57)	1.43 (0.84‒2.44)	
**Drinking**							0.231
No	**Ref**	1.22 (0.43‒3.48)	1.26 (0.60‒2.66)	1.14 (0.67‒1.95)	0.88(0.51‒1.53)	1.60 (0.93‒2.76)	
Yes	**Ref**	1.01 (0.58‒1.76)	1.86 (1.08‒3.20)*	1.18 (0.83‒1.67)	1.47 (1.03‒2.10)^c^	1.95 (1.36‒2.79)^a^	
**Smoke**							0.221
Current smoker	**Ref**	0.68 (0.30‒1.54)	1.27 (0.58‒2.78)	1.08 (0.70‒1.67)	1.02 (0.64‒1.62)	1.66 (1.06‒2.61)^c^	
Former smoker	**Ref**	0.88 (0.31‒2.49)	1.29 (0.55‒3.03)	1.00 (0.52‒1.93)	1.20 (0.63‒2.29)	1.36 (0.73‒2.54)	
Never smoker	**Ref**	1.69 (0.80‒3.59)	2.61 (1.28‒5.34)^b^	1.40 (0.83‒2.36)	1.70 (0.98‒2.96)	2.92 (1.75‒4.86)^a^	

Note: ‘^a^’ 0.001 ‘^b^’ 0.01 ‘^c^’ 0.05.

**Fig. 3:**
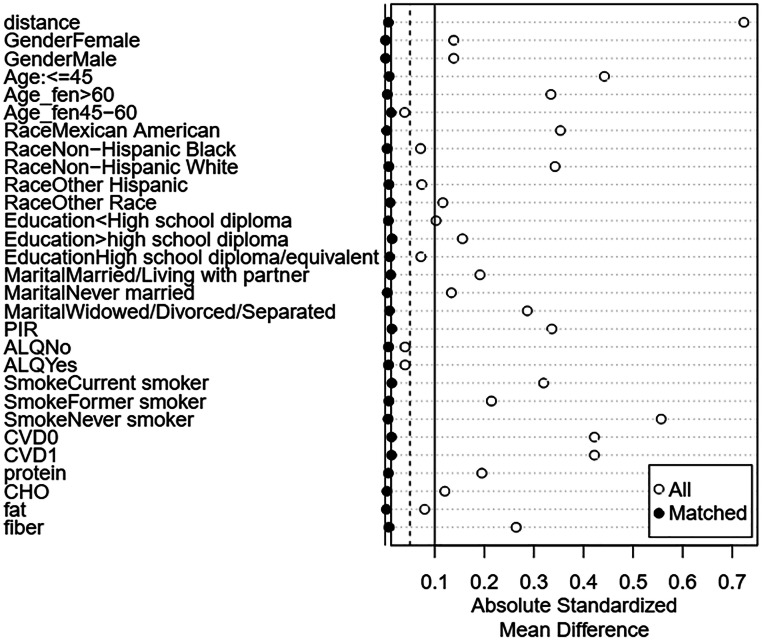
To minimize confounding bias from covariates and further examine the relationship of COPD with MHO and MUO phenotypes and BMI.

PSM was conducted to minimize confounding bias from covariates and further examine the association of COPD with MHO and MUO phenotypes and BMI (Fig. 3). After PSM, in contrast to the MH—NW individuals, the MUO individuals were associated with significantly higher prevalence of COPD among three models (Model 1: OR = 1.94, 95% CI 1.43–2.64; Model 2: OR = 1.86, 95% CI 1.36–2.54; Model 3: OR = 1.87, 95% CI 1.37–2.54; p < 0.001; Supplementary Table S1). When treated as a continuous variable, BMI was positively associated with the prevalence of COPD, and this association achieved statistical significance among three models (Model 1: OR = 1.03, 95% CI 1.03–1.04; Model 2: OR = 1.03, 95% CI 1.03–1.04; Model 3: OR = 1.04, 95% CI 1.03–1.04; p < 0.001; Supplementary Table S2). Further comparisons across BMI categories indicated that, regardless of model adjustment, obesity was associated with significantly elevated prevalence of COPD (Model 1: OR = 1.55, 95% CI 1.34–1.78; Model 2: OR = 1.56, 95% CI 1.35–1.79; Model 3: OR = 1.59, 95% CI 1.39–1.82; p < 0.001; Supplementary Table S2), whereas overweight was not significantly with the prevalence of COPD (Model 1: OR = 1.01, 95% CI 0.87–1.18; Model 2: OR = 1.03, 95% CI 0.88–1.20; Model 3: OR = 1.05, 95% CI 0.90–1.22; p > 0.05; Supplementary Table S2). After excluding CVD patients and metabolically healthy obese individuals (redefined using only biochemical indicators), self-reported diagnoses and medication use, consistent results were noted (Supplementary Tables S3 and S4). After excluding individuals with hypertension and diabetes, the results were still consistent (Supplementary Tables S5 and S6).

## Discussion

The authors analyzed data from 28,401 U.S. adults in the NHANES dataset to examine the association of BMI and MHO and MUO phenotypes with the prevalence of COPD. The present results demonstrated a significant association between MUO and the prevalence of COPD. Further analysis revealed that MUO individuals were associated with a higher prevalence of COPD compared with MH—NW individuals. Multivariable analyses demonstrated that even after adjustments for confounders such as sex and age, the prevalence of COPD remained significantly higher among MUO individuals than MH—NW individuals. These findings highlight the strong relevance of metabolic health status to the prevalence of COPD. RCS analyses further suggested a nonlinear association between BMI and COPD. BMI demonstrated a linear association with COPD among metabolically healthy individuals. Moreover, ROC curve analysis indicated that BMI exhibited relatively high diagnostic ability for COPD among the metabolically healthy individuals. By contrast, in the metabolically unhealthy group, BMI exhibited a nonlinear association with the odds of having COPD, with a threshold BMI value at 28.733 kg/m^2^. The estimated inflection point reflects a model-based turning point, rather than a precise biological or diagnostic threshold, and its apparent numerical accuracy should not be over-interpreted. These results further confirmed the complex nature of the association between BMI and COPD.

The findings regarding the association between BMI and COPD remain inconsistent. A study has suggested that obesity may be associated with more severe COPD progression.^5^ Conversely, other studies found no significant effect of obesity on the likelihood of COPD exacerbation.^15^^,^^16^ Furthermore, several studies suggest a negative association between obesity and all-cause mortality among hospitalized COPD and community-based populations, suggesting the presence of a potential obesity paradox in obstructive lung diseases.^17^ Given that obesity is frequently concurrent with such metabolic abnormalities as hyperglycemia, dyslipidemia, and hypertension, this study classified the selected metabolic disorder indicators and explored the differential association between metabolic phenotypes and COPD. The results demonstrated that after excluding potential confounders, MUO individuals had significantly elevated odds of having COPD. RCS analyses in the metabolically healthy group suggested a nonlinear association between BMI and the odds of COPD, particularly showing increased odds when BMI reached 30.468 kg/m^2^. In contrast, the association between BMI and the odds of COPD among the metabolically unhealthy individuals appeared linear, further supporting the influence of BMI as a potential risk marker for COPD.

This study reveals that metabolic abnormalities may have a more significant impact on the prevalence of COPD than overweight or obesity alone. This finding is consistent with previous research results. Some studies also suggest that the comorbidity of Metabolic Syndrome (MetS) and COPD may exacerbate the disease progression and prognosis.^18^^,^^19^ In the potential linkage mechanism between COPD and MetS, systemic inflammation and oxidative stress may play important roles.^20^ Existing cross-sectional studies and some longitudinal studies suggest that the higher prevalence of MetS in COPD patients may be associated with elevated systemic inflammation levels, and this association may further affect the progression of respiratory function and the trajectory of lung function.^10^^,^^11^^,^^21^ The pro-inflammatory properties of oxidative stress may be one of the potential pathways linking COPD and MetS-related cardiovascular complications. However, its specific mechanism of action still needs to be verified through a more rigorous experimental design. In addition, COPD patients usually have reduced physical activity capacity, which further increases a series of health problems, including comorbidities. The coexisting MetS further reduces the physical activity of COPD patients.^22^ Furthermore, due to the lack of physical activity, patients with MetS may experience reduced lung function and accelerated progression of COPD.^23^

Interaction tests showed that age had a significant interaction effect on the association between the metabolic obesity phenotype and COPD. Age-stratified subgroup analyses revealed that MUO was significantly associated with elevated prevalence of COPD in younger and middle-aged adults (< 60-years), whereas no such association was found in the older population (≥ 60-years). It has been reported[23] that insulin resistance is more pronounced in younger and middle-aged individuals, which may impair lung function, increase the risk of COPD, and lead to a more active immune system response. In MUO, adipose tissue may participate in the systemic inflammatory response of COPD by secreting inflammatory cytokines such as TNF-α and IL-6, thereby increasing the risk of the disease.^24^ However, the relevant mechanisms still need to be further validated in experiments. Some younger and middle-aged MUO individuals may also engage in unhealthy lifestyle behaviors, including smoking and physical inactivity.^25^ These adverse behaviors, together with MUO, may be jointly associated with a higher prevalence of COPD. With advancing age, lifestyle patterns of older adults tend to change, as reflected by a decline in smoking prevalence and a shift toward moderate exercise, which may partly mitigate the influence of MUO on COPD risk. Additionally, older individuals are often prescribed long-term medications for multiple comorbidities. For instance, corticosteroids, the most frequently used anti-inflammatory agents in COPD management,^26^ may exert regulatory effects on pulmonary function and interfere with MUO-related metabolic processes, thereby complicating the assessment of COPD risk. Multi-subgroup analyses may increase the risk of type I errors, thus increasing the likelihood that some statistically significant findings may occur by chance. Although age showed a significant interaction effect, the present study was an exploratory analysis, and multiple tests were not corrected for. Therefore, these results should be interpreted with caution.

This study is based on the NHANES database, which is nationally representative, contains large sample sizes, and uses rigorous weighted analysis methods. Its complex sampling design ensures that the data can be extrapolated to the entire US population. Continuous collection of large sample sizes provides ample statistical power for the study. The accompanying weighting guidelines guide researchers in performing complex sampling corrections to obtain unbiased and accurate parameter estimates, thus ensuring the scientific validity of the results.

It is essential to consider several limitations of this study when interpreting these findings. Firstly, causal relationships between BMI and COPD cannot be inferred because this research applied a cross-sectional design. Secondly, the present study did not obtain detailed information on the disease course of COPD, which may be a crucial factor influencing changes in BMI during the study period. Thirdly, the use of self-reported data could cause recall bias, particularly regarding COPD diagnosis and classification variables. It is particularly important to note that criteria based on medication history may not be effective in distinguishing between COPD and asthma-COPD overlap syndrome, potentially leading to misclassification bias. The possibility of asthma-COPD overlap and its potential impact on the present study results should be considered. The use of medications was also used as an inclusion criterion. While it is necessary to maintain sample size and statistical power, it introduces a significant risk of misclassification, particularly for individuals with asthma or asthma-COPD overlap. The authors are unable to perform a sensitivity analysis using only the gold-standard spirometry data, and thus, the results should be interpreted with caution. Finally, unmeasured confounding variables may exist that could affect the study outcomes. As this is an exploratory analysis, these findings should be interpreted with caution and need to be validated in longitudinal studies and randomized controlled trials.

## Conclusion

MUO is associated with an increased prevalence of COPD among U.S. adults. Regardless of metabolic health status, obesity is associated with an elevated prevalence of COPD. Future studies could employ longitudinal designs to assess whether weight or metabolic management in MUO individuals reduces the incidence of COPD.

## Clinical trial number

Not applicable.

## Ethics approval and consent to participate

The NHANES data are de-identified and publicly available; therefore, this secondary analysis was exempt from institutional ethics review.

## Consent for publication

Not applicable.

## Data availability

The datasets used and analysed during the current study are available from the corresponding author on reasonable request.

## Funding

This work was supported by the Anrotinine combined with Ampurimumab/Anrotinine combined chemotherapy/chemical first-line treatment for patients with late-term lung cancer, single central heart, side-to-side control bed therapeutic effect TB213014. And the Dazhou Central Hospital Institute-level Scientific Research Project 2024YJ04.

## Declaration of competing interest

The authors declare no conflicts of interest.

## Appendix Supplementary materials

Image, application 1

## Footnotes

Supplementary material associated with this article can be found, in the online version, at doi:10.1016/j.clinsp.2026.101006.
