# Supplementary material for: Relationship between metabolically unhealthy obesity and chronic obstructive pulmonary disease among U.S. adults: Evidence from NHANES 1999–2018
Source: Clinics (Sao Paulo). 2026 Jul 17;81:101006. doi: 10.1016/j.clinsp.2026.101006 (PMC13401014; doi:10.1016/j.clinsp.2026.101006)
Supplement: Supplementary file 1 [file mmc1.docx]

CLINICS-D-25-01704_Supplementary Material

**Table S1** The association between COPD and Metabolically Healthy Obesity according to different variables.

| **Participants** | **Model 1** | **Model 2** | **Model 3** |
| --- | --- | --- | --- |
|  | **OR (95% CI)** | **OR (95% CI)** | **OR (95% CI)** |
| MHO |  |  |  |
| MH-NW | Ref. | Ref. | Ref. |
| MH-OW | 1.00 (0.59‒1.68) | 1.01 (0.60‒1.69) | 1.02 (0.61‒1.73) |
| MHO | 1.54 (0.92‒2.57) | 1.56 (0.94‒2.60) | 1.59 (0.96‒2.63) |
| MU-NW | 1.30 (0.95‒1.78) | 1.23 (0.89‒1.69) | 1.20 (0.87‒1.64) |
| MU-OW | 1.29 (0.95‒1.75) | 1.24 (0.91‒1.69) | 1.24 (0.91‒1.68) |
| MUO | 1.94 (1.43‒2.64)^a^ | 1.86 (1.36‒ 2.54)^a^ | 1.87 (1.37‒2.54)^a^ |

Note: ^a^’ 0.001 ‘**’ 0.01 ‘*’ 0.05.

Model 1: NO covariate has been adjusted. Model 2: Adjusted Gender + Age grouping + Race + Education. Model 3: Adjusted Gender + Age grouping + Race + Education + Marital + PIR + Dringking + Smoke. MH-NW, Metabolism Healthy Normal Weight; MH-OW, Metabolism Healthy Overweight; MHO, Metabolism Healthy Obesity; MU-NW, Metabolism Unhealthy Normal Weight; MU-OW, Metabolism Unhealthy Overweight; MUO, Metabolism Unhealthy Obesity.

**Table S2** Relationship between BMI and COPD after PSM.

| **Participants** | **Model 1** | **Model 2** | **Model 3** |
| --- | --- | --- | --- |
|  | **OR (95% CI)** | **OR (95% CI)** | **OR (95% CI)** |
| BMI |  |  |  |
| Continuous | 1.03 (1.03‒1.04)^a^ | 1.03(1.03‒1.04)^a^ | 1.04 (1.03‒1.04)^a^ |
| Grouping |  |  |  |
| Normal weight | ref. | ref. | ref. |
| Overweight | 1.01 (0.87‒1.18) | 1.03 (0.88‒1.20) | 1.05 (0.90‒1.22) |
| Obesity | 1.55 (1.34‒1.78)^a^ | 1.56 (1.35‒1.79)^a^ | 1.59 (1.39‒1.82)^a^ |

Note: ‘^a^’ 0.001 ‘**’ 0.01 ‘*’ 0.05.

Model 1: NO covariate has been adjusted. Model 2: Adjusted Gender + Age grouping + Race + Education. Model 3: Adjusted Gender + Age grouping + Race + Education + Marital + PIR + Dringking + Smoke.

**Table S3** Relationship between BMI and COPD (Exclude CVD).

| **Participants** | **Model 1** | **Model 2** | **Model 3** |
| --- | --- | --- | --- |
|  | **OR (95% CI)** | **OR (95% CI)** | **OR (95% CI)** |
| BMI |  |  |  |
| Continuous | 1.03 (1.03‒1.04)^a^ | 1.03 (1.03‒1.04)^a^ | 1.04 (1.03‒1.05)^a^ |
| Grouping |  |  |  |
| Normal weight | Ref. | Ref. | Ref. |
| Overweight | 1.06 (0.89‒1.27)^c^ | 1.10 (0.92‒1.31) | 1.17 (0.98‒1.38) |
| Obesity | 1.59 (1.37‒1.84)^a^ | 1.61 (1.39‒1.87)^a^ | 1.71 (1.46‒2.01)^a^ |
|  |  |  |  |
| **MHO** |  |  |  |
| **MH-NW** | Ref. | Ref. | Ref. |
| **MH-OW** | 1.02 (0.61‒1.71) | 1.05 (0.64‒1.73) | 1.12 (0.68‒1.85) |
| **MHO** | 1.73 (1.07‒2.80)^c^ | 1.81 (1.13‒2.92)^c^ | 1.85 (1.15‒2.98)^c^ |
| **MU-NW** | 1.69 (1.25‒2.29)^a^ | 1.35 (0.99‒1.84) | 1.18 (0.88‒1.58) |
| **MU-OW** | 1.69 (1.25‒2.29)^a^ | 1.45 (1.06‒1.99)^c^ | 1.36 (1.00‒1.86) |
| **MUO** | 2.42 (1.77‒3.30)^a^ | 2.05 (1.49‒2.83)^a^ | 1.95 (1.43‒2.67)^a^ |

Note: ‘^a^’ 0.001 ‘**’ 0.01 ‘^c^’ 0.05.

Model 1: NO covariate has been adjusted. Model 2: Adjusted Gender + Age grouping + Race + Education. Model 3: Adjusted Gender + Age grouping + Race + Education + Marital + PIR + Dringking + Smoke.

**Table S4** Relationship between BMI and COPD (Metabolically unhealthy is redefined using only biochemical markers, excluding self-reported diagnoses and medication use).

| **Participants** | **Model 1** | **Model 2** | **Model 3** |
| --- | --- | --- | --- |
|  | **OR (95% CI)** | **OR (95% CI)** | **OR (95% CI)** |
| BMI |  |  |  |
| Continuous | 1.02 (1.01‒1.04)^a^ | 1.03 (1.02‒1.04)^a^ | 1.03 (1.02‒1.04)^a^ |
| Grouping |  |  |  |
| Normal weight | Ref. | Ref. | Ref. |
| Overweight | 1.14 (0.91‒1.42) | 1.19(0.96‒1.49) | 1.27 (1.01‒1.58)^c^ |
| Obesity | 1.57 (1.27‒1.94)^a^ | 1.70 (1.36‒2.13)^a^ | 1.77 (1.40‒2.22)^a^ |
|  |  |  |  |
| **MHO** |  |  |  |
| **MH-NW** | Ref. | Ref. | Ref. |
| **MH-OW** | 1.12 (0.66‒1.89) | 1.13 (0.68‒1.87) | 1.25 (0.74‒2.08) |
| **MHO** | 1.96 (1.21‒3.20)^c^ | 2.07 (1.28‒3.35)^b^ | 2.11 (1.30‒3.42)^b^ |
| **MU-NW** | 1.419 (1.00‒1.97)^c^ | 1.15 (0.83‒1.61) | 1.04 (0.76‒1.43) |
| **MU-OW** | 1.54 (1.10‒2.17)^c^ | 1.389 (0.98‒1.95) | 1.32 (0.93‒1.85) |
| **MUO** | 1.98 (1.37‒2.85)^a^ | 1.83 (1.27‒2.65)^a^ | 1.75 (1.22‒2.51)^b^ |

Note: ‘^a^’ 0.001 ‘^b^’ 0.01 ‘^c^’ 0.05.

Model 1: NO covariate has been adjusted. Model 2: Adjusted Gender + Age grouping + Race + Education Model 3: Adjusted Gender + Age grouping + Race + Education + Marital + PIR + Dringking + Smoke.

**Table S5** Relationship between BMI and COPD (exclude hypertension).

| **Participants** | **Model 1** | **Model 2** | **Model 3** |
| --- | --- | --- | --- |
|  | **OR (95% CI)** | **OR (95% CI)** | **OR (95% CI)** |
| BMI |  |  |  |
| Continuous | 1.04 (1.02‒1.05)^a^ | 1.04 (1.03‒1.05)^a^ | 1.04 (1.03‒1.05)^a^ |
| Grouping |  |  |  |
| Normal weight | Ref. | Ref. | Ref. |
| Overweight | 1.17 (0.92‒1.50) | 1.22 (0.95‒1.55) | 1.25 (0.98‒1.60) |
| Obesity | 1.72 (1.38‒2.16)^a^ | 1.80 (1.43‒2.27)^a^ | 1.77 (1.40‒2.26)^a^ |
|  |  |  |  |
| **MHO** |  |  |  |
| **MH-NW** | Ref. | Ref. | Ref. |
| **MH-OW** | 0.95 (0.57‒1.57) | 0.94 (0.58‒1.53) | 1.03 (0.63‒1.67) |
| **MHO** | 1.69 (1.07‒2.68)^c^ | 1.76 (1.12‒2.78)^c^ | 1.76 (1.12‒2.78)^c^ |
| **MU-NW** | 1.19 (0.87‒1.64) | 1.02 (0.74‒1.40) | 0.93 (0.69‒1.25) |
| **MU-OW** | 1.47 (1.06‒2.02)^c^ | 1.36 (0.98‒1.89) | 1.26 (0.90‒1.75) |
| **MUO** | 1.99 (1.41‒2.81)^a^ | 1.84 (1.30‒2.61)^a^ | 1.67 (1.19‒2.35)^b^ |

Note: ‘^a^’ 0.001 ‘^b^’ 0.01 ‘^c^’ 0.05.

Model 1: NO covariate has been adjusted. Model 2: Adjusted Gender + Age grouping + Race + Education. Model 3: Adjusted Gender + Age grouping + Race + Education + Marital + PIR + Dringking + Smoke.

**Table S6** Relationship between BMI and COPD (exclude diabetes).

| **Participants** | **Model 1** | **Model 2** | **Model 3** |
| --- | --- | --- | --- |
|  | **OR (95% CI)** | **OR (95% CI)** | **OR (95% CI)** |
| BMI |  |  |  |
| Continuous | 1.03 (1.02‒1.04)^a^ | 1.03 (1.02‒1.04)^a^ | 1.03 (1.02‒1.04)^a^ |
| Grouping |  |  |  |
| Normal weight | Ref. | Ref. | Ref. |
| Overweight | 0.97 (0.79‒1.19) | 0.99 (0.81‒1.21) | 1.06 (0.87‒1.28) |
| Obesity | 1.46 (1.23‒1.75)^a^ | 1.52 (1.27‒1.81)^a^ | 1.58 (1.33‒1.89)^a^ |
|  |  |  |  |
| **MHO** |  |  |  |
| **MH-NW** | Ref. | Ref. | Ref. |
| **MH-OW** | 0.95 (0.57‒1.57) | 0.98 (0.60‒1.58) | 1.05 (0.65‒1.71) |
| **MHO** | 1.69 (1.07‒2.68)^c^ | 1.76 (1.12‒2.77)^c^ | 1.75 (1.12‒2.74)^c^ |
| **MU-NW** | 2.32 (1.71‒3.15)^a^ | 1.70 (1.23‒2.36)^a^ | 1.45 (1.06‒1.99)^c^ |
| **MU-OW** | 1.88 (1.38‒2.56)^a^ | 1.56 (1.11‒2.18)^c^ | 1.45 (1.04‒2.02)^c^ |
| **MUO** | 2.62 (1.96‒3.50)^a^ | 2.21 (1.63‒3.00)^a^ | 2.07 (1.54‒2.78)^a^ |

Note: ‘^a^’ 0.001 ‘**’ 0.01 ‘^c^’ 0.05.

Model 1: NO covariate has been adjusted. Model 2: Adjusted Gender + Age grouping + Race + Education. Model 3: Adjusted Gender + Age grouping + Race + Education + Marital + PIR + Dringking + Smoke.
